# Analysis of PNGase F‐Resistant N‐Glycopeptides Using SugarQb for Proteome Discoverer 2.1 Reveals Cryptic Substrate Specificities

**DOI:** 10.1002/pmic.201700436

**Published:** 2018-06-10

**Authors:** Johannes Stadlmann, David M. Hoi, Jasmin Taubenschmid, Karl Mechtler, Josef M. Penninger

**Affiliations:** ^1^ Institute of Molecular Biotechnology Austrian Academy of Sciences Dr. Bohr Gasse 3 A‐1030 Vienna Austria; ^2^ Institute of Molecular Pathology Campus‐Vienna‐Biocenter 1 A‐1030 Vienna Austria

## Abstract

SugarQb (http://www.imba.oeaw.ac.at/sugarqb) is a freely available collection of computational tools for the automated identification of intact glycopeptides from high‐resolution HCD MS/MS datasets in the Proteome Discoverer environment. We report the migration of SugarQb to the latest and free version of Proteome Discoverer 2.1, and apply it to the analysis of PNGase F‐resistant N‐glycopeptides from mouse embryonic stem cells. The analysis of intact glycopeptides highlights unexpected technical limitations to PNGase F‐dependent glycoproteomic workflows at the proteome level, and warrants a critical reinterpretation of seminal datasets in the context of N‐glycosylation‐site prediction.

Glycosylation, the covalent attachment of simple or complex carbohydrate structures onto proteins, is one of the most abundant post‐translational modification (PTM), and affects virtually all aspects of life.^1^ Over 50% of human proteins are predicted to carry these important and dynamic sugar modifications, which alter their activities in fundamental biological processes, such as intracellular trafficking, cell adhesion, signal transduction, essential immune functions, or host–pathogen interactions.^2^ In contrast to other PTMs, glycosylation remains largely unexplored at the proteome scale. Despite the massive technological advances in mass‐spectrometry (MS)‐based proteomics, the enormous structural complexity and the rather unfavorable fragmentation properties of intact glycopeptides still pose a formidable challenge to the concurrent analysis of the peptide and glycan moieties by tandem mass‐spectrometry (MS/MS).

Consequently, pioneering studies in the field of glycoproteomics primarily focused on the identification of enzymatically de‐N‐glycosylated peptides by MS/MS.^3^, ^4^ To this end, glycopeptides were first specifically enriched using a wide range of techniques (e.g., lectins,^4^ titanium dioxide,^5^ or via hydrazone formation of periodate oxidized carbohydrate cis‐diol groups^6^) and then, prior to LC‐MS/MS analysis, subjected to enzymatic de‐glycosylation. This key reaction, catalyzed by peptide‐N4‐(N‐acetyl‐beta‐glucosaminyl) asparagine amidases (i.e., PNGases), results in the specific cleavage of N‐glycans from the polypeptide‐backbone and thus allows to identify the former N‐glycopeptides as non‐glycosylated peptides. Additionally, the enzymatic hydrolysis of the N‐glycosidic bond results in the deamidation of formerly N‐glycosylated asparagine residues. This PNGase induced conversion of asparagine to aspartic acid results in a mass increment (i.e., 0.984 amu), and has been suggested to provide means for the specific localization of N‐glycosylation sites.^7^

Although these seminal studies were intrinsically limited to the analysis of enzymatically de‐N‐glycosylated peptides, they greatly contributed to our current knowledge of N‐glycosylation site occupancy within the proteome and provided the basis for many advanced N‐glycosylation‐site prediction algorithms. For example, using this approach in a single large‐scale study, more than 6000 N‐glycosylation sites within the murine proteome have been mapped site specifically.^4^ Importantly, however, early biochemical characterizations of PNGases also reported on subtle substrate requirements of these enzymes, particularly with respect to the primary structure of substrate N‐glycopeptides.^8^, ^9^, ^10^ More specifically, the key enzyme PNGase F has been reported of not being able to remove N‐glycans from peptide‐N‐ and peptide‐C‐terminal asparagine residues.^8^, ^9^ As these long‐standing observations suggest important technical limitations to the comprehensive characterization of the N‐glycoproteome in PNGase F‐dependent workflows, we were prompted to evaluate their impact on the analysis of the N‐glycoproteome by identifying and analyzing PNGase F‐resistant N‐glycopeptides using the recently developed SugarQb platform.^11^

Aiming at a comprehensive characterization of intact glycopeptides from complex samples, we recently developed a collection of data interpretation tools, which allows for the automated identification of intact glycopeptides from high‐resolution HCD MS/MS datasets, using well‐established proteomic MS/MS search engines (e.g., MASCOT, SEQUEST‐HT, MS Amanda^12^). SugarQb (http://www.imba.oeaw.ac.at/sugarqb) analyses MS/MS spectra for the presence of potential [peptide + HexNAc]+ fragment ions. For this, the mass of the respective precursor ion is iteratively reduced by the masses represented in a user‐defined glycan mass database (Table S1, Supporting Information), −203.0794 amu. In cases where a corresponding potential [peptide + HexNAc]+ fragment ion is detected the respective spectra are duplicated, with the original precursor ion mass being set to the mass of the potential [peptide + HexNAc]+ fragment ion. Subsequently, the preprocessed MS/MS spectra are searched using commonly used MS/MS search engines for peptide sequence identification. In addition to this core functionality, SugarQb also provides a range of other computational tools for the automated identification of glycopeptide MS/MS spectra (i.e., G‐score), charge deconvolution, and de‐isotoping (i.e., MS2 spectrum processor), as well as the specific removal of highly abundant glycan‐derived fragment ions (i.e., Reporter Ion Filter). SugarQb is freely available as Node to the Proteome Discoverer Platform and thus readily integrated into typical shot gun proteomic data interpretation workflows. It allows for taking advantage of modern MS instrumentation (i.e., high‐resolution and high‐mass accuracy mass analyzers, high sensitivity, high speed in data acquisition), quantitative proteomic tools (e.g., isotope encoded labeling techniques), and the retrospective analysis of untargeted MS/MS datasets. Additionally, we now also migrated SugarQb to the new, freely available Proteome Discoverer 2.1 platform (https://portal.thermo-brims.com/).

To identify potentially PNGase F‐resistant N‐glycopeptides, we first generated tryptic digests from whole cell lysates of mouse embryonic stem cells (mESC), desalted them using SPE C18 cartridges and subjected them to enzymatic de‐glycosylation by incubation with 1 U of PNGase F (from *Elizabethkingia miricola*) per milligram peptide in 200 mM Tris/HCl, pH 8.0, at 37 °C for 18 h. Then, we enriched the remaining glycopeptides using IP‐HILIC and analyzed them by RP‐nLC‐ESI‐MS/MS^11^, ^13^ using stepped collision energy HCD (i.e., SCE‐HCD, using 35% NCE +/−5%) on an Orbitrap Fusion LUMOS instrument.^14^ The MS/MS data were preprocessed and analyzed as reported previously,^11^ using the SugarQb platform in the Proteome Discoverer 2.1 environment in conjunction with the two MS/MS search engines MASCOT and MS Amanda,^12^ searching the Uniprot mouse reference proteome set (UP00000589, release‐2016_08, 47 435 entries; as concatenated forward and reverse database; Figure 1A). In this study, for all MS/MS search engines, the proteolytic cleavage rules were set to those of trypsin, allowing for up to two missed cleavage sites. Carbamidomethylation of cysteines was set as fixed modification, and the oxidation of methionine was considered as variable modification. Additionally, all asparagine, serine and threonine side chains could be variably modified with a single hexosamine residue. The precursor mass tolerance was set to 10 ppm, the fragment mass tolerance was set to 25 mmu. Amino acid sequence identification was based on matching singly‐charged b‐ and y‐fragment ion series, considering ammonia and water losses, as well as the neutral loss of HexNAc.^11^ The resulting peptide spectrum matches (PSMs) were manually prefiltered (i.e., best scoring search engine rank 1 PSMs only, peptide length greater than six amino acids), sorted by the respective search engine score value and then filtered to 1% FDR using the concatenated forward and decoy approach.^15^ Site localization of N‐glycans was performed using ptmRS.^16^

**Figure 1:**
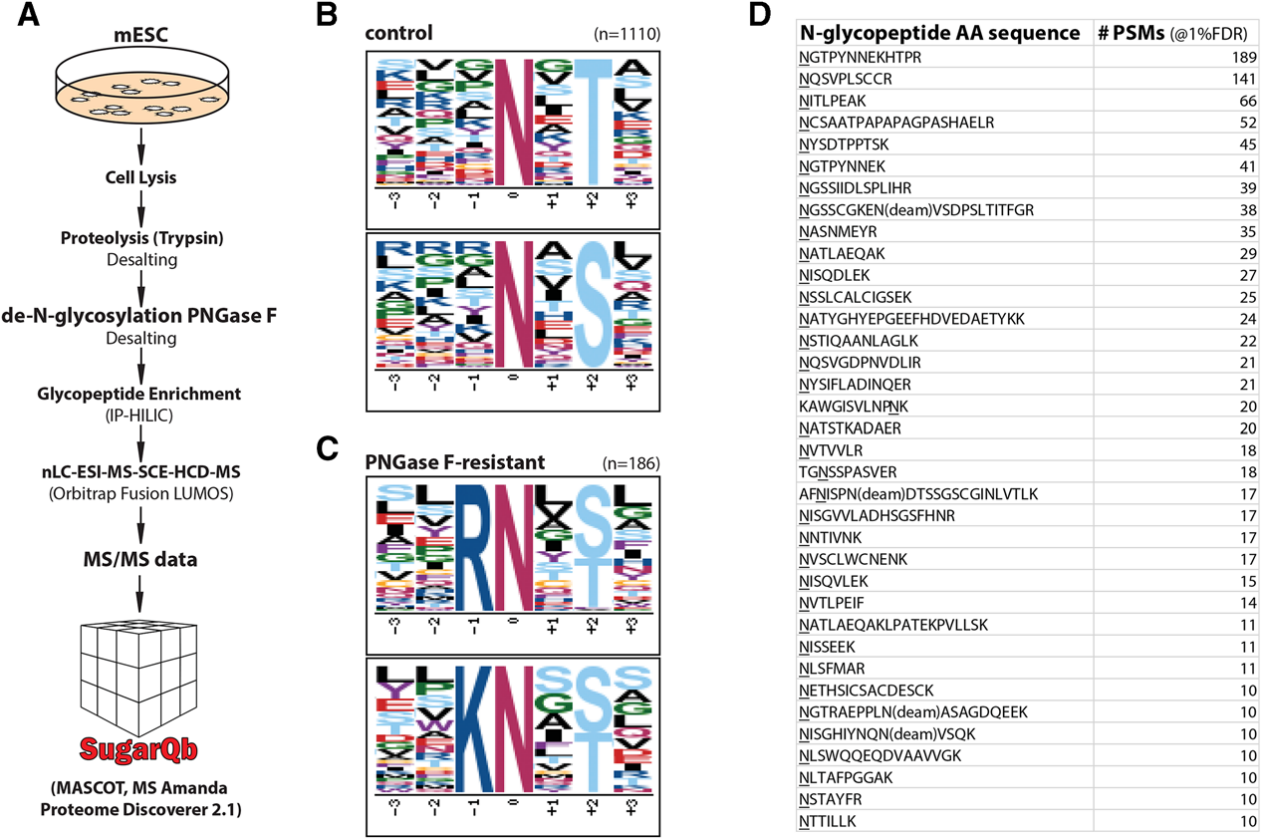
A) Workflow for the analysis of PNGase F‐resistant N‐glycopeptides, using the SugarQb platform in the Proteome Discoverer 2.1 environment. B) Sequence motif analysis of 1110 unique N‐glycopeptide sequences of the untreated control sample confirms the specific enrichment of the N‐glycosylation motif N‐!P‐S/T. C) Motif analysis corroborates N‐terminal, glycosylated asparagine residues of tryptic glycopeptides as “PNGase F‐resistant.” D) PNGase F‐treatment results in the specific enrichment of intact N‐glycopeptide spectrum matches (PSMs), exhibiting N‐terminal, glycosylated asparagine residues. All experiments shown have been performed in duplicate, with very similar results.

The analysis of the PNGase F‐treated samples led to the identification of 365 and 242 unique, glycosylated peptide sequences, using MASCOT and MS Amanda, respectively (Tables S2 and S3, Supporting Information). Surprisingly, despite extensive enzymatic de‐N‐glycosylation of the samples, next to 183 O‐glycosylated glycopeptides, we also identified the amino acid sequences of 186 “PNGase F‐resistant,” intact N‐glycopeptides, using MASCOT. Of note, this compares to 1110 N‐glycopeptide sequences identified by MASCOT in the control samples, which were not treated with PNGase F (Table S4, Supporting Information). MS Amanda was performed similarly (i.e., 1047 N‐glycopeptide sequences identified; Table S5, Supporting Information). Detailed amino acid sequence analysis of “PNGase F‐resistant” tryptic N‐glycopeptides (using “motif‐x”^17^), revealed them to predominantly bear N‐terminal N‐glycosylated asparagine residues (i.e., 131 of 186 N‐glycopeptide sequences; 50‐fold enrichment of the sequence motif K/R‐N‐!P‐S/T over the control sample as background; Figure 1B–D), corroborating previously reported substrate specificities of PNGase F.^8^, ^9^ Importantly, we did neither observe selective depletion nor enrichment of specific glycans on PNGase F‐resistant peptides. This suggests that PNGase F‐resistance was largely independent of the N‐glycan structures attached.

To more precisely quantify the susceptibility of the N‐glycoproteome to PNGase F‐treatment, we used a comparative glycoproteomic approach.^11^ For this, we labeled tryptic digests of mESC whole cell lysates with TMT‐6plex (Thermo; 200 mg of tryptic peptides per TMT channel), desalted and treated them with increasing amounts of PNGase F (i.e., 0, 0.5 U and 5 U PNGase F mg^−1^ protein in 200 mM Tris/HCl, pH 8.0) for 18 h at 37 °C. After incubation, the individual samples were adjusted to pH 2 by the addition of 10% formic acid, pooled and desalted using SPE C18 cartridges. Eventually PNGase F‐resistant peptides were enriched using IP‐HILIC (Figure 2A).

**Figure 2:**
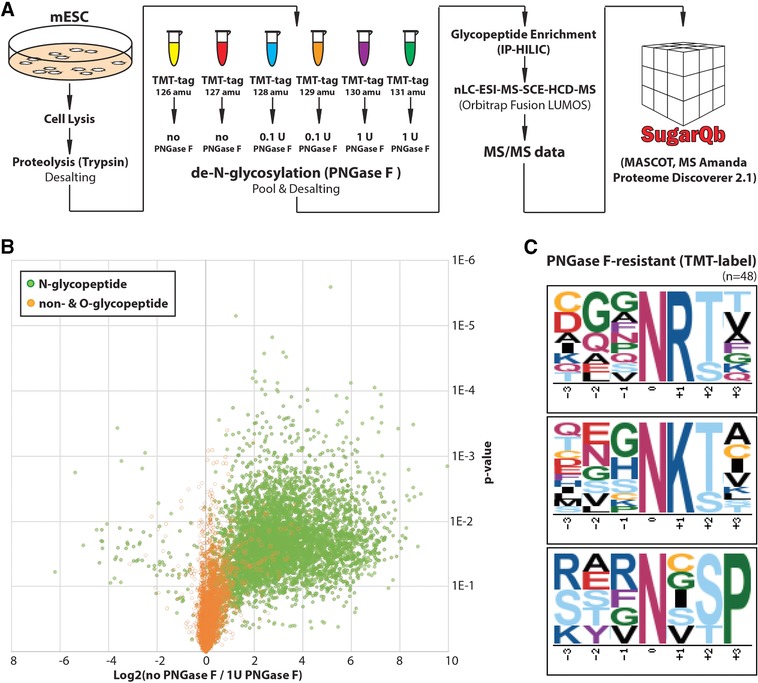
A) Comparative glycoproteomic workflow quantifying the sensitivity of N‐glycopeptides to PNGase F‐treatment, using the SugarQb platform in Proteome Discoverer 2.1. B) Volcano plot of the comparative glycoproteomic dataset shows a large population of N‐glycopeptides (green) to be sensitive to the incubation with 1 U PNGase F (i.e., 5 U PNGase F mg^−1^ protein). Non‐glycosylated peptides and O‐glycopeptides (orange) were not sensitive to PNGase F‐treatment. Of note, a small population of multiple N‐glycosylated peptides became more abundant upon PNGase F‐treatment. C) Motif analysis of TMT‐labeled, PNGase F‐resistant N‐glycopeptide sequences reveals glycosylated asparagine in the penultimate position of tryptic glycopeptides and those being part of the consensus sequence N‐!P‐S/T‐P as poor substrates. All experiments shown have been performed in duplicate, with very similar results.

From our subsequent analysis by RP‐nLC‐ESI‐MS/MS, we identified and comparatively quantified 985 glycopeptide sequences upon PNGase F‐treatment (Tables S6 and S7, Supporting Information). In contrast to the previous, TMT label‐free experiment, we did not observe an important resistance of TMT‐labeled N‐terminally N‐glycosylated peptides. Indeed, 5 U PNGase F mg^−1^ protein were able to remove N‐glycans from N‐terminal asparagine residues of TMT‐labeled N‐glycopeptides to a large extent (Figure 2B). In contrast to historically important dabsyl‐ or dansyl‐labelling,^9^ NHS‐ester‐based labelling of amino‐terminal primary amine groups reconstitutes amide bonds, N‐terminally to the glycosylated asparagine residues. We speculate that the presence of this additional amide bond effectively abolishes the PNGase F‐resistance of this glycopeptide population, warranting further experiments using alternative amine reactive labeling reagents (e.g., other NHS esters, or organic acid anhydrides).

Furthermore, the quantitative glycoproteomic data highlighted N‐glycosylated asparagine residues, which lie at the penultimate position of tryptic glycopeptides, to be poor substrates for PNGase F (eightfold enrichment of the sequence motif N‐K/R‐S/T over the control sample as background; Figure 2C). Despite extensive PNGase F‐treatment, at least 80% of their initial abundance was recovered. Of note, since the use of trypsin results in the accumulation of lysine and arginine at the c‐terminal end of glycopeptides, a clear delineation of the specific impact of these positively charged amino acids on PNGase F activity necessitates further investigations of non‐tryptic N‐glycopeptides. Sequence‐specific differences in the susceptibility to the enzymatic deglycosylation are further highlighted by the observation of N‐glycopeptide variants which become more abundant upon PNGase F‐treatment (Figure 2B). This intriguing glycopeptide population consists of N‐glycopeptides which carry more than one N‐glycan. PNGase F‐resistance of one of these multiple N‐glycosylation sites resulted in the increased abundance of partially deglycosylated N‐glycopeptides identified in our analysis. Also, in the comparative glycoproteomic experiments, we did not observe selective depletion or enrichment of specific N‐glycan species found on PNGase F‐resistant peptides.

Importantly, our analysis also revealed N‐glycopeptide sequences, which comprise the established N‐glycosylation motif, followed by proline (i.e., N‐!P‐S/T‐P), to be highly resistant to the deglycosylation by PNGase F. This observation sheds new light on previously published glycoproteomics data. For example, Zielinska et al.,^4^ analyzing the amino acid sequences of 6367 N‐glycosylation sites of the PNGase F‐sensitive mouse N‐glycoproteome, also reported an unexpected depletion of proline in position 4 in the mouse glycoproteome. Similarly, Kaji et al.,^18^ analyzing 1495 PNGase A‐sensitive N‐glycosylation sites from *Caenorhabditis elegans*, merely identified a single site comprising the motif N‐!P‐S/T/C‐P. Based on our analysis of intact N‐glycopeptides from mESC, we could not only confirm the existence of N‐glycosylated peptides comprising this motif, but also provide a rational for their apparent depletion in large‐scale PNGase F‐dependent glycoproteomic datasets.

In summary, we here report on the analysis of intact N‐glycopeptides from mESC, which are insensitive to the enzymatic deglycosylation with different concentrations of PNGase F. Our analyses were performed using the recently developed SugarQb platform within the freely available Proteome Discoverer 2.1 environment. The results of our analysis of intact glycopeptides highlight subtle technical limitations intrinsic to PNGase F‐dependent glycoproteomic workflows at the proteome level, and warrant a reinterpretation of these seminal datasets in the context of N‐glycosylation site prediction.

## Conflict of Interest

The authors declare no conflict of interest.

## Supporting information

Supporting information.

Supporting information.

Supporting information.

Supporting information.

Supporting information.

Supporting information.

Supporting information.
